# Effectiveness of mRNA BNT162b2 Vaccine 6 Months after Vaccination among Patients in Large Health Maintenance Organization, Israel

**DOI:** 10.3201/eid2802.211834

**Published:** 2022-02

**Authors:** Jennifer Kertes, Sharon Baruch Gez, Yaki Saciuk, Lia Supino-Rosin, Naama Shamir Stein, Miri Mizrahi-Reuveni, Anat E. Zohar

**Affiliations:** Maccabi HealthCare Services, Tel Aviv‒Jaffa Israel

**Keywords:** COVID-19, coronavirus disease, severe acute respiratory syndrome coronavirus 2, SARS-CoV-2, coronaviruses, viruses, respiratory infections, zoonoses, serologic tests, vaccines, mRNA BNT162b2 vaccine, vaccination, epidemiology, public health, infections, immune response, health maintenance organization, HMO, Israel

## Abstract

Israel experienced a new wave of coronavirus disease during June 2021, six months after implementing a national vaccination campaign. We conducted 3 discrete analyses using data from a large health maintenance organization in Israel to determine whether IgG levels of fully vaccinated persons decrease over time, describe the relationship between IgG titer and subsequent PCR-confirmed infection, and compare PCR-confirmed infection rates by period of vaccination. Mean IgG levels steadily decreased over the 6-month period in the total tested population and in all age groups. An inverse relationship was found between IgG titer and subsequent PCR-positive infection. Persons vaccinated during the first 2 months of the campaign were more likely to become infected than those subsequently vaccinated. The vaccinated group >60 years of age had lower initial IgG levels and were at greater risk for infection. The findings support the decision to add a booster vaccine for persons >60 years of age.

Coronavirus disease (COVID-19) was identified in Israel at the end of February 2019 (*1*). As in other countries, Israel has experienced several infection waves. The third wave, largely attributed to entry of the Alpha virus variant into Israel, began during in September 2020; at its peak, >8,000 new cases were being identified daily (*2*). Israel was among the first countries to introduce a national vaccination campaign using the mRNA BNT162b2 vaccine (Pfizer-BioNTech, https://www.pfizer.com). The BNT162b2 vaccine received emergency approval for use by the US Food and Drug Administration after the vaccine showed 95% efficacy over an average 2-month follow-up period (*3*,*4*). The vaccine was initially approved for any person >16 years of age, with a recommended 21-day interval, 2-dose administration.

The vaccine campaign began on December 20, 2020 (concurrent with a 2-month nationwide lockdown), first targeting all healthcare workers and the population >60 years of age and quickly extending to all persons >16 years of age. Initially, those persons who had a previous infection were not eligible for vaccination, but within 3 months, policy was changed to offer a single dose to all persons who had a previous infection. By April 2021, >50% of persons >16 years of age and 88% of persons >50 years of age countrywide had been fully vaccinated (*2*). The number of new cases decreased to 140 cases/day by April 2021 (*2*). Initial population-based studies in Israel comparing vaccinated and unvaccinated groups reported vaccine effectiveness rates of 95% (*5*,*6*).

One of the biggest questions regarding the vaccine is the length of protection provided. In publishing third-phase research results, Pfizer-BioNTech reported a 91% efficacy rate over a 6-month follow-up period and an estimated 6% decrease in efficacy every 2 months (*7*). Population-based observational studies in Israel are no longer a feasible method of evaluating long-term effectiveness of the vaccine, given that most persons have now been fully vaccinated. Infection rates in Israel increased again during June‒September 2021 (fourth wave), and most (97%) positive cases were infected with the Delta variant (B.1.617.2) (G. Rahib, Israel Ministry of Health Laboratories, pers. comm., 2021 Aug 8). Initial serologic studies of the Delta variant suggest that the BNT162b2 vaccine provides protection against Delta variant infection, but at lower rates than for the Alpha variant (88% vs. 93.7%) (*8*). Given the increase in infection rates, the dilemma arose whether this increase was attributable to reduced effectiveness of the vaccine against the Delta variant or a waning of protection provided by the vaccine over time.

The objective of this study was to determine if the BNT162b2 vaccine had become less effective in preventing infection, and if so, in which population groups and to what degree. To meet this objective, we conducted 3 discrete analyses to answer the following questions. First, do antibody levels (IgG) of those fully vaccinated decrease over time and if so, for who and how quickly? Second, what is the relationship between antibody level (IgG) and subsequent PCR-confirmed infection? Third, is there a difference in PCR-confirmed infection incidence rates between persons vaccinated in the initial months of the vaccination campaign and persons vaccinated later?

## Methods

We conducted a series of retrospective cohort analyses to meet the study objectives. We extracted all data from the Maccabi Healthcare Services database (https://www.maccabi4u.co.il/1781-he/Maccabi.aspx). Maccabi is the second-largest health maintenance organization (HMO) in Israel and provides healthcare coverage for >2.5 million citizens (27% of the population of Israel). The database includes demographic data (date of birth, sex, socioeconomic status based on census, and national survey classifications applied to home address); laboratory data (all PCR and IgG test results); and health status data (chronic illness registries, such as heart disease, hypertension, chronic kidney disease [CKD], diabetes and immunosuppressive disorder, based on hospital and community-based diagnoses and procedures, and relevant laboratory and test results). The study was approved by the Maccabi Helsinki Committee (#0178-20-MHS). Informed consent was waived because all data extracted from the database were anonymized and aggregated.

### Testing Procedures

PCR testing is conducted free of charge for any HMO member who has symptoms or reported exposure to a confirmed case. Testing is conducted by using real-time reverse transcription PCR (Allplex 2019-nCoV Assay; Seegene Inc., https://www.seegene.com). We offered serologic testing to specific target populations, such as employees (19%) and residents and employees of geriatric medical and retirement home facilities owned by the HMO (4%) at discrete points in time, but most (77%) testing was carried out in the general HMO population for whom testing is freely available upon request (patients initiative). We conducted IgG testing by using severe acute respiratory syndrome coronavirus 2 (SARS-CoV-2) spike-specific antibodies and a follow-up chemo-luminescence immunoassay (Quant II IgG anti-Spike CoV2-SARS; Abbott Laboratories, https://www.abbott.com) and reported as arbitrary units per milliliter (AU/mL). Antibody levels are reported numerically, except for outliers (<21 AU/mL and >40,000 AU/mL), which are coded. Coded results were converted to numeric results (<21 to 21 and >40,000 to 40,001).

### IgG Levels of Vaccinated Population over Time

All HMO members who had received both vaccine doses and had a subsequent IgG test for SARS-CoV-2 antibodies >7 days after the second vaccination were included in this component of the study. The study period extended from January 11, 2021 (when those first vaccinated reached day 7 after the second dose), through July 7, 2021. We mapped IgG results over a 180-day period by using demographic and health characteristics (age group, sex, socioeconomic status, and presence of selected chronic illnesses).

### Relationship between IgG Levels and Subsequent SARS-CoV-2 Infection

We included all HMO members who had a PCR test (irrespective of vaccination status) during June 1‒July 14, 2021 (peak of fourth wave of infection), and an IgG serologic test 7‒120 days before the PCR test in this component of the study. We used the most recent test result for persons who had >1 test. We used the most recent PCR test date if all results were negative and the date of the first positive PCR result for persons who had >1 result. We calculated the proportion of participants who had subsequent positive PCR results by antibody level status.

### Comparison of Infection Rates by Vaccination Period

We included all HMO members who as of June 9, 2021, were >7 days post‒second vaccination dose and had no previous positive PCR result in this component of the study. We excluded from analysis members who received 3 doses or had an appointment to receive the third dose (n = 320) during the follow-up period. (At this time, a recommendation to offer a booster vaccination for persons who had an immune-suppressive disorder had been authorized.) We categorized the study population by using vaccination completion: January‒February 2021 and March‒May 2021. For both groups, we calculated the proportion who were PCR positive during June 9‒July 18, 2021 (yes/no).

### Statistical Analyses

We used Mann‒Whitney and Kruskal‒Wallis tests to compare antibody levels over time between different population groups. We used linear regression to identify those factors associated with serologic levels. Natural logarithm (ln) of serologic levels showed a normal distribution and was selected as the outcome variable. Other variables we entered into a hierarchical model were days from vaccination; age, sex, and socioeconomic status; and selected chronic illnesses.

We used χ^2^ analyses to test the association between serologic levels (categorized) and PCR outcomes. We categorized serologic status into <300 AU/mL or >300 AU/mL We calculated Kaplan‒Meier survival curves to compare time from serologic test to positive PCR result for the serologic categories by using log-rank tests. We defined an event as a positive PCR result. Time to event was the number of days from a serologic test to PCR, with censoring for those who died, left the HMO, or had a follow-up period of <120 days. We used logistic regression analysis to compare PCR-positive outcomes between vaccination periods, while controlling for age group, socioeconomic status, and presence of chronic illness (heart disease, hypertension, diabetes, CKD, and immunosuppressive disorder).We performed statistical analyses by using SPSS Statistics 25 (https://www.ibm.com) and R version 3.6.2 (https://cran.r-project.org).

## Results

### IgG Levels of Vaccinated Population over time

The study population consisted of 8,395 persons (Table 1). Of all HMO members who received both vaccine doses, those subsequently tested for IgG were more likely to be male, younger (18–44 years of age), and in a higher socioeconomic bracket and less likely to have a chronic illness than those not tested for IgG.

**Table 1:** Demographic and health characteristics of population vaccinated against coronavirus disease, by serologic test status, Maccabi Healthcare Services, Israel, January‒July 2021

Characteristic	No. (%) not tested, n = 1,423,257	No. (%) tested, n = 8,395
Sex		
M	683,946 (48.1)	2,774 (33.0)
F	739,311 (51.9)	5,621 (67.0)
Age group, y		
<18	39,123 (2.7)	92 (1.1)
18–44	644,038 (45.3)	2,199 (26.2)
45–59	384,100 (27.0)	3,016 (35.9)
60–74	255,377 (17.9)	2,515 (30.0)
>75	100,619 (7.1)	573 (6.8)
Socioeconomic status		
Low	481,470 (33.8)	3,202 (38.1)
Middle	232,824 (16.4)	1,281 (15.3)
High	708,963 (49.8)	3,912 (46.6)
Heart disease		
No	1,346,990 (94.6)	7,790 (92.8)
Yes	76,267 (5.4)	605 (7.2)
Diabetes		
No	1,297,140 (91.1)	7,342 (87.5)
Yes	126,117 (8.9)	1,053 (12.5)
Hypertension		
No	1,145,327 (80.5)	5,960 (71.0)
Yes	277,930 (19.5)	2,435 (29.0)
Chronic kidney disease		
No	1,353,406 (95.1)	7,715 (91.9)
Yes	69,851 (4.9)	680 (8.1)
Immunosuppressive disorder		
No	1,396,529 (98.1)	7,411 (88.3)
Yes	26,728 (1.9)	984 (11.7)

**Figure 1:**
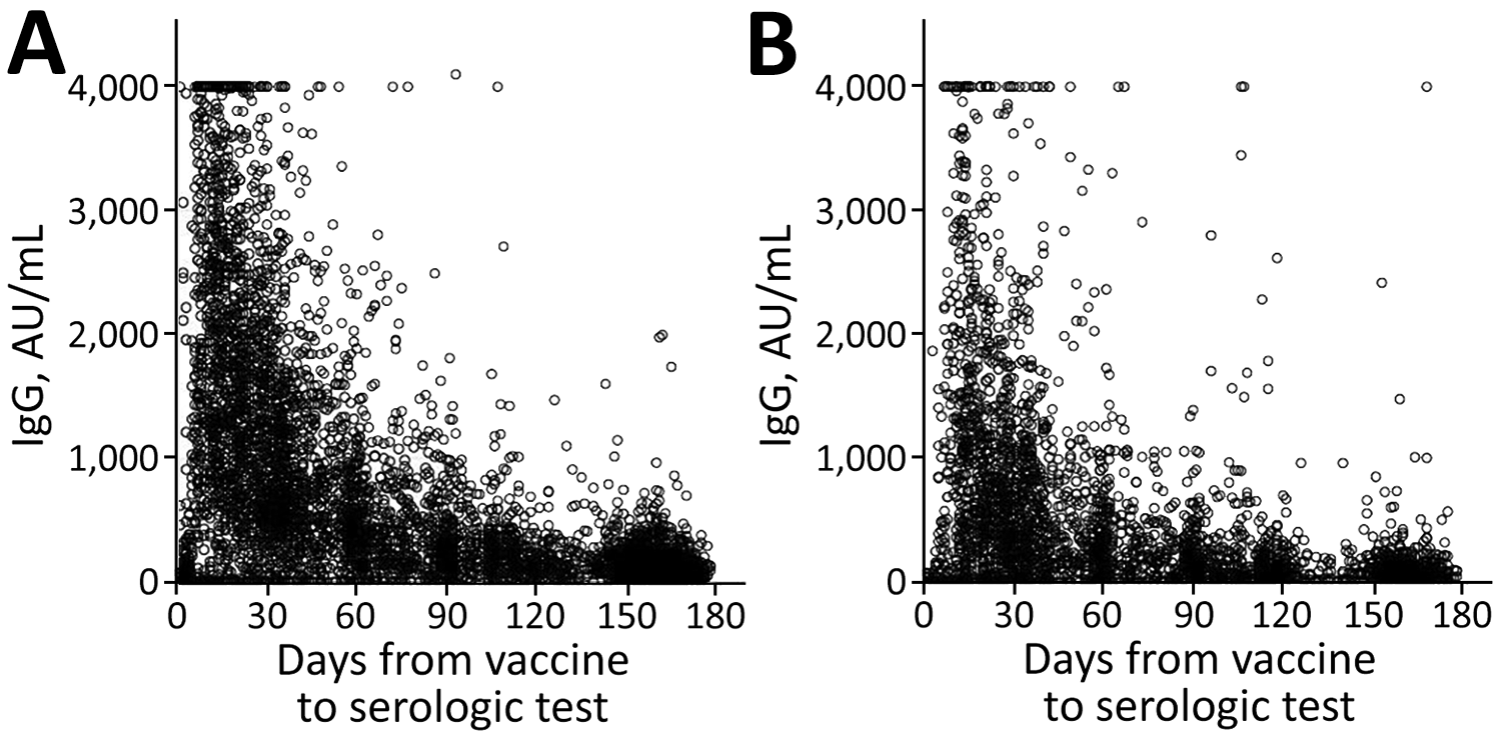
IgG levels for population vaccinated with mRNA BNT162b2 vaccine (Pfizer-BioNTech, https://www.pfizer.com) against coronavirus disease over time, by age group, Maccabi Healthcare Services, Israel, January‒June 2021. A) <60 years of age. B) >60 years of age.

We found that serologic levels in the study population decreased over time, from a mean of 14,008 for those tested within a month of being vaccinated to a mean of 1,411 for those tested in the sixth month after vaccination (Table 2). We observed a decrease over time in all subpopulation groups when results were stratified by age group, sex, socioeconomic status, and selected chronic illnesses (Table 2). The largest mean differences between subpopulations were observed in their initial serologic levels (within the first month). Mean serologic levels for participants >60 years of age (n = 1,004, mean 9,433) were approximately half of those for participants <60 years of age (n = 1,453, mean 17,169) in the first month, attenuating to a <10% difference 6 months later (Figure 1). Large differences in initial serologic levels were also observed for participants with chronic illness, in particular participants with an immunosuppressive disorder, CKD or heart disease. Initial (first month) serologic levels increased by socioeconomic level.

**Table 2:** Mean antibody level by demographic and health variables and time from vaccination against coronavirus disease for population vaccinated against coronavirus disease, Maccabi Healthcare Services, Israel, January‒July 2021

Characteristic	Days from vaccination to serologic test						p value
7–29	30–59	60–89	90–119	120–150	>150
Total population	2,457	1,845	946	827	500	1,820	
Antibody levels							
Mean	14,008	8,175	4,365	2,706	1,773	1,411	<0.001
SD	12,146	7,742	5,022	3,957	1,934	1,751	
Median	11,322	6,080	2,974	1,683	1,217	1,217	
Sex							
No. male	1,075	676	354	269	105	295	
Mean antibody level	12,278	6,837	3,799	2,633	1,695	1,309	<0.001
No. female	1,382	1,169	592	558	395	1,525	
Mean antibody level	15,354	8,949	4,703	2,740	1,794	1,431	
Age group, y							
No. <18	32	40	14	6	0	0	
Mean antibody level	29,781	15,348	9,971	9,421			<0.001
No. 18–44	677	469	262	201	142	448	
Mean antibody level	18,522	9,866	5,621	3,271	2,006	1,479	
No. 45–59	744	602	288	237	224	921	
Mean antibody level	15,396	8,875	4,279	2,793	1,929	1,419	
No. 60–74	821	599	302	264	99	430	
Mean antibody level	9,999	6,280	3,684	2,670	1,478	1,256	
No. >75	183	135	80	119	35	20	
Mean antibody level	6,892	5,468	2,147	1,316	668	912	
Socioeconomic status							
No. low	553	278	143	113	59	135	
Mean antibody level	15,994	10,048	4,481	4,056	2,443	1,625	<0.001
No. middle	1,088	827	414	392	259	932	
Mean antibody level	13,989	8,473	4,739	2,523	1,695	1,468	<0.001
No. H\high	816	740	389	322	182	753	
Mean antibody level	12,687	7,139	3,924	2,454	1,667	1,301	<0.001
Underlying conditions							
Heart disease							
No. patients	206	165	79	75	25	55	
Mean antibody level	7,341	4,307	2,520	2,455	690	1,575	<0.001
Diabetes							
No. patients	377	245	121	123	57	130	
Mean antibody level	8,624	6,647	2,742	2,189	843	1,401	<0.001
Hypertension							
No. patients	803	572	274	290	133	363	
Mean antibody level	9,930	6,624	3,032	2,118	1,341	1,409	<0.001
Chronic kidney disease							
No. patients	248	163	81	88	45	55	
Mean antibody level	6,756	4,331	2,614	2,339	887	1,910	<0.001
Immunosuppressive disorder							
No. patients	307	280	156	126	57	58	
Mean antibody level	6,824	4,371	2,336	1,500	1,033	1,813	<0.001

Of all persons who were vaccinated with both doses, 2.8% also had a positive PCR result. Comparable decreases in serologic means by month were observed in this group, as in others. However, the mean serologic level for those tested in the first 7–30 days was much higher than that for the total study population (22,630 AU/mL; p<0.001).

When demographic and health variables were entered into a linear regression model (Appendix Table 1), all factors remained independently associated with serologic levels; the highest coefficient was observed for participants who had an immunosuppressive disorder. No multicollinearity was observed between the factors in the regression model.

### Relationship between IgG Levels and Subsequent SARS-CoV-2 Infection

Demographic and health characteristics of HMO members who had a serologic result and who had subsequently been tested by PCR were similar to those who had no PCR test result (Table 3), with the exception of socioeconomic status and diabetes status. Persons who were tested by PCR were more likely to be in the lower socioeconomic bracket and have diabetes than persons not tested by PCR.

**Table 3:** Demographic and health characteristics of population vaccinated against coronavirus disease, by PCR test status, Maccabi Healthcare Services, Israel, January‒July 2021

Characteristic	No. (%) not tested, n = 79,404	No. (%) tested, n = 5,141
Sex		
M	33,871 (42.7)	2,128 (41.4)
F	45,533 (57.3)	3,013 (58.6)
Age group, y		
<18	21,563 (27.2)	937 (18.2)
18–44	37,470 (47.2)	2,338 (45.5)
45–59	13,553 (17.1)	1,090 (21.2)
60–74	5,672 (7.1)	657 (12.8)
>75	1,146 (1.4)	119 (2.3)
Socioeconomic status		
Low	10,744 (13.5)	1,339 (26.0)
Middle	36,358 (45.8)	1,234 (24.0)
High	32,302 (40.7)	2,568 (50.0)
Heart disease		
No	78,105 (98.4)	5,021 (97.7)
Yes	1,299 (1.6)	120 (2.3)
Diabetes		
No	76,320 (96.1)	4,903 (95.4)
Yes	3,084 (3.9)	238 (4.6)
Hypertension		
No	73,063 (92.0)	4,524 (88.0)
Yes	6,341 (8.0)	617 (12.0)
Chronic kidney disease		
No	78,058 (98.3)	5,012 (97.5)
Yes	1,346 (1.7)	129 (2.5)
Immunosuppressive disorder		
No	78,193 (98.5)	5,010 (97.5)
Yes	1,211 (1.5)	131 (2.5)

Of persons who had both serologic and PCR tests (n = 5,141), 57% had a serologic test result of <150 AU/mL, 6% had a result of 150‒299 AU/mL, 10% had a result of 300–799 AU/mL, and 27% had a result >800 AU/mL. The proportion of participants with a positive PCR result were 1.2% for those who had serologic levels <150 AU/mL, 1.3% for those who had serologic levels of 150–299 AU/mL, 0.2% for those who had serologic levels of 300–799 AU/mL, and 27% for those who had serologic levels of >800 AU/mL (p = 0.004). Mean serologic levels for the 42 study participants who had a positive PCR result were 175 AU/mL (SD +490 AU/mL) compared a mean serologic level of 2,057 AU/mL (SD +6,030 AU/mL) for those with a negative result (p<0.001).

**Figure 2:**
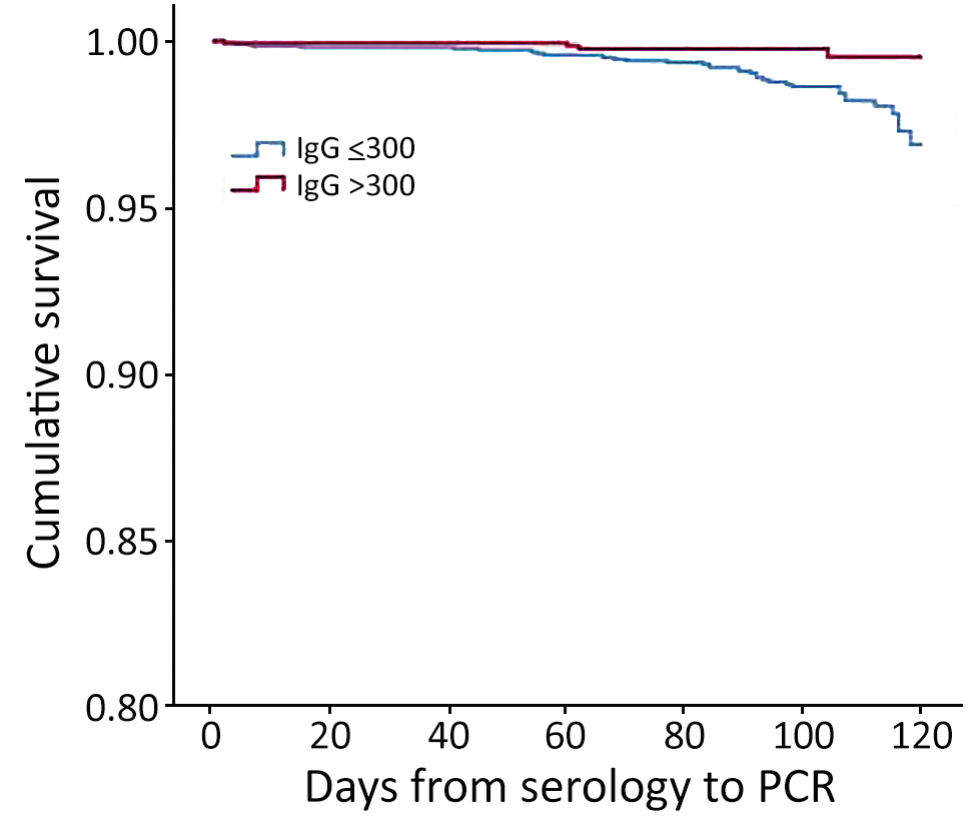
Kaplan-Meier cumulative survival for PCR-positive outcome for population vaccinated with mRNA BNT162b2 vaccine (Pfizer-BioNTech, https://www.pfizer.com) against coronavirus, by antibody (IgG) level, Maccabi Healthcare Services, Israel, June‒July 2021.

Of all the study participants for this component of the study, 365 (7%) had a previous infection (37% of whom received 1 vaccine dose). Those who had a previous infection were less likely to have a serologic level of <300 AU/mL than those who did not have a previous infection (40.3% vs. 65.2%; p<0.001). However, irrespective of previous infection (yes/no), the proportion of those with a new PCR positive result was 0.8%. We provide Kaplan‒Meier survival curves (over time by serologic status and +300 AU/mL) (Figure 2). The curves indicate that participants who had lower serologic levels (<300 AU/mL) had lower survival rates than participants who had higher serologic levels (+300 AU/mL; p = 0.03 by log-rank test).

### Comparison of Infection Rates by Vaccination Period

At the time of the study, 86% of those eligible for vaccination (>16 years of age; n = 1,423,098) had received both doses in the HMO (90% of those >60 years of age). We compared demographic and health variables between those who were vaccinated in the first 2 months with those vaccinated later (Table 4). Those who were vaccinated in the first 2 months were more likely to be older, in a higher socioeconomic bracket, and have higher rates of chronic illness. We found that 1,518 (0.19%) of those vaccinated during January‒February 2021 were PCR positive compared with 644 (0.11%) of those vaccinated during March‒May 2021 (p<0.001). Univariate analyses (Appendix Table 2) also showed that age, sex, socioeconomic status and presence of chronic illnesses (health disease, diabetes, hypertension, and CKD) were associated with having a positive PCR result.

**Table 4:** Demographic and health characteristics of population vaccinated against coronavirus disease, by vaccination period, Maccabi Healthcare Services, Israel, January‒July 2021

Characteristic	Jan‒Feb, no. (%), n = 821,231	Mar‒May, no. (%), n = 601,867
Sex		
M	394,546 (48.0)	285,089 (47.6)
F	426,685 (52.0)	313,482 (52.4)
Age group, y		
<18	21,232 (2.6)	62,793 (10.4)
18–44	211,351 (25.7)	414,514 (68.9)
45–59	289,813 (35.3)	86,013 (14.3)
60–74	219,437 (26.7)	28,620 (4.8)
>75	79,398 (9.7)	9,927 (1.6)
Socioeconomic status		
Low	102,689 (12.5)	125,924 (20.9)
Middle	398,474 (48.5)	307,724 (51.1)
High	320,068 (39.0)	168,219 (27.9)
Heart disease		
No	755,979 (92.1)	592,608 (98.5)
Yes	65,252 (7.9)	9,259 (1.5)
Diabetes		
No	717,950 (87.4)	581,893 (96.7)
Yes	103,281 (12.6)	19,974 (3.3)
Hypertension		
No	593,368 (72.3)	556,545 (92.5)
Yes	227,863 (27.7)	45,322 (7.5)
Chronic kidney disease		
No	762,717 (92.9)	592,447 (98.4)
Yes	58,514 (7.1)	9,420 (1.6)
Immunosuppressive disorder		
No	808,061 (98.4)	598,499 (99.4)
Yes	13,170 (1.6)	3,368 (0.6)

Factors associated with subsequent infection (positive PCR result) in a logistic regression model (Table 5) were socioeconomic status, age group, vaccination period, sex, and heart disease. When controlling for all other factors, we found that members vaccinated first were 1.6 times more likely to get infected with COVID-19 than those vaccinated later.

**Table 5:** Factors associated with coronavirus disease for population vaccinated against coronavirus disease, by logistic regression model, Maccabi Healthcare Services, Israel, January‒July 2021

Variable	No.	Adjusted odds ratio (95% CI)
Vaccination period		
Jan‒Feb	821,231	1.61 (1.45–1.79)
Mar‒May	601,867	Referent
Sex		
M	681,382	0.11 (1.01–1.20)
F	741,716	Referent
Age group, y		
<18	84,025	Referent
18–44	625,865	1.92 (1.51–2.49)
45–59	375,826	1.88 (1.46–2.45)
60–74	248,057	1.54 (1.17–2.04)
.>75	89,325	1.06 (0.75–1.50)
Socioeconomic status		
Low	228,613	Referent
Moderate	706,198	2.85 (2.34–3.50)
High	488,287	4.40 (3.61–5.41)
Heart disease		
No	1,348,587	Referent
Yes	74,511	1.35 (1.11–1.79)
Diabetes		
No	1,299,843	Referent
Yes	123,255	1.03 (0.87–1.12)
Hypertension		
No	1,149,913	Referent
Yes	273,185	0.98 (0.86–1.22)
Chronic kidney disease		
No	1,355,164	Referent
Yes	67,934	0.82 (0.63–1.05)
Immunosuppressive disorder		
No	1,406,596	Referent
Yes	16,502	0.90 (0.58–1.63)

## Discussion

In this study, we found that IgG serologic levels for SARS-CoV-2 virus decreased progressively over time for the total vaccinated population and in each subpopulation when stratified by demographic and health variables. We also found an association between serologic levels and subsequent risk for infection, wherein participants who had a serologic level <300 AU/mL were more likely to get COVID-19 than those who had a serologic level >300 AU/mL. We established that those vaccinated at the beginning of the national vaccination campaign were more likely to get infected (during the current wave of infection) than those vaccinated later. These findings suggest that effectiveness of the vaccine decreases over time and that the current wave of infection can be attributed, at least in part, to the reduced effectiveness of the vaccine over time.

Initial serologic studies focused on patients found to be PCR positive for COVID-19 reported a decrease over time of antibody presence from time of infection (*9**–**11*). Fewer studies have looked specifically at serologic response of the vaccinated population. Most of the studies based on vaccinated populations reported ≈100% seroconversion rates but had short follow-up periods (*12*,*13*). Serologic levels were much higher among the vaccinated population than those convalescing after infection (*12*) and among those <50 years of age (*13*). In a case‒control study of PCR-positive case-patients divided by previous vaccination status (yes/no), Lopez-Bernal et al. (*8*) found that those vaccinated (2 doses) with the BNT162b2 vaccine and were infected with the Alpha variant achieved 93.7% vaccine effectiveness rate compared with an 88% vaccine effectiveness rate for those infected with the Delta variant.

We did not find published studies that described serologic status over longer follow-up periods for a vaccinated population. Mean levels of IgG decreased progressively over time for all subpopulations in this study. The difference between the groups was mostly evident in initial (first month) starting means; the elderly and those having chronic illness had lower levels, but these levels attenuated to more comparable levels between groups 6 months after vaccination. In a large household study in the United Kingdom (*14*), IgG response measured over the first 3 months after vaccination found higher seroconversion rates for younger age groups (20–40 years of age), female participants, those receiving both doses, vaccination with BNT162b2 vaccine compared with AstraZeneca (https://www.astrazeneca.com) vaccine, and those with evidence of a previous infection. Low responders were older and had higher prevalence of chronic illness/disease, such as patients receiving immune suppressants or who had diabetes. These same population groups were found in this study to start with lower serologic levels and have lower mean serologic levels 6 months after vaccination.

One of the many unknowns regarding COVID-19 is to what extent IgG is indicative of protection against the virus. The manufacturer’s recommended cutoff indicating a positive serologic response (<50 AU/mL) is much lower than the mean serologic levels we found at 6 months after vaccination. Are higher levels indicative of higher protection? Other mechanisms of protection, such as antiviral T and B cell memory, have been suggested as offering protection, even in the absence of seroconversion (*15*). In a meta-analysis, Khoury et al. (*16*) found a strong relationship between mean neutralization levels and reported protection. They further estimated that protection was likely to occur over 250 days, although with still largely preserved protection from severe infection. In the second component of our study, we found an association between serologic level and PCR outcome in which increased serologic level was associated with decreasing infection rates. Using a cutoff value of 300 AU/mL, we found higher rates of infection for those with low serologic levels. However, given that those coming for testing were not randomly selected, repeat studies in a large randomly selected population are required to confirm this cutoff value.

Few data are available to compare vaccine effectiveness over time, and observational follow-up studies are becoming less appropriate, given the potential bias between those electing to vaccinate and those who do not. Pfizer-BioNTech published a recent efficacy study that compared symptomatic infection rates between vaccinated and unvaccinated groups over a period of 6 months (*7*). Vaccine efficacy for infection decreased from 96% within the first 2 months post‒second dose to 84% vaccine efficacy 4‒6 months post‒second dose. Consistent with the findings of Pfizer-BionTech, we found higher rates of COVID-19 infection among those vaccinated in the initial months of the vaccine campaign compared with those vaccinated later. Even after controlling for age (those vaccinated first were more likely to be older), incidence rates were higher in the first vaccinated group. Were most of the fourth wave of infections attributable to the Delta virus, we would have expected consistent incidence rates, irrespective of when the individual was vaccinated. We suggest that the difference found here between time periods indicates a reduction in vaccine effectiveness over time. However, we cannot rule out some contribution of the Delta variant to reduced effectiveness.

One limitation of our study was that test findings were not based on repeated tests in the same population but a description of the results over time of those coming for a serologic test. Those coming for serologic and PCR testing were not randomly selected groups but, rather, persons volunteering in a study or, more commonly, requesting to be tested. Participants requesting a test (serologic and PCR) might have had greater concerns regarding exposure, infection, or perceived infection risk, potentially increasing the proportion of persons who had lower serologic levels or a positive PCR result. We calculated mean serologic levels for each subpopulation, despite the potential for outlier measures to skew results, to enable statistical comparison between subpopulation groups. Numbers were small for some stratified data, particularly for the 120–149 day period, and should be interpreted with caution. Study findings were not adjusted for serologic test accuracy. Conclusions are made on the assumption that most of those infected in the third component of the study (by time of vaccination) were infected with the Delta variant, given its prevalence in Israel.

All data presented are for Maccabi Healthcare Service members. Maccabi members are more likely to come from a higher socioeconomic bracket, and the service has a somewhat larger prevalence of members 35–55 years of age than that for the total population of Israel (*17*). Although these differences would not affect stratified data in our study, these differences might effect mean serologic results for the total population and vaccine effectiveness results. Generalizability of results to Israel and other countries should be made cautiously.

Given these limitations, the different elements of the study were based on large numbers of a vaccinated population who had 6 months of follow-up time to measure COVID-19 infection. We found that serologic levels for all groups decreased over time and that there was an association between serologic levels and subsequent infection. We further observed that persons vaccinated early in the vaccination campaign had higher infection rates. These factors taken together suggest that the BNT162b2 vaccine, as indicated by the manufacturer, offers lower protection against infection over time, independent of SARS-CoV-2 variant type. These results contributed to the decision to offer a third dose of the BNT162b2 vaccine to persons >60 years of age. Follow-up of infection and illness rates in this group will enable us to confirm the wisdom of providing a booster dose.

Appendix Additional information on effectiveness of mRNA BNT162b2 vaccine 6 months after vaccination among patients in large health maintenance organization, Israel.
