# Supplementary material for: Effectiveness of mRNA BNT162b2 Vaccine 6 Months after Vaccination among Patients in Large Health Maintenance Organization, Israel
Source: Emerg Infect Dis. 2022 Feb;28(2):338–46. doi: 10.3201/eid2802.211834 (PMC8798683; doi:10.3201/eid2802.211834)
Supplement: Appendix — Additional information on effectiveness of mRNA BNT162b2 vaccine 6 months after vaccination among patients in large health maintenance organization, Israel. [file 21-1834-Techapp-s1.pdf]

Article DOI: https://doi.org/10.3201/eid2802.211834

Effectiveness of mRNA BNT162b2 Vaccine 6 Months after Vaccination among Patients in Large Health Maintenance Organization, Israel

Appendix

Appendix Table 1. Factors associated with natural logarithm of serologic titers by linear regression model*

Factor	B	p value
Days from vaccination	–0.016	<0.0001
Male sex	–0.347	<0.0001
Age, y	–0.022	<0.0001
Socioeconomic status (middle)	–0.078	0.033
Socioeconomic status (high)	–0.184	<0.0001
Heart disease	–0.242	<0.0001
Diabetes	–0.108	0.007
Hypertension	–0.105	0.001
Chronic kidney disease	–0.318	<0.0001
Immunosuppressive disorder	–-0.888	<0.0001

*R^2 = 0.498. Dependent variable is ln of serologic titer. Independent variables entered in hierarchical model: days from vaccination; sex, age, and socioeconomic status; chronic illnesses.

Appendix Table 2. Factors associated with a positive PCR result by univariate analysis

Factor	No.	No. positive (%)	p value*
Sex			<0.01
M	681,382	1,096 (0.16)	
F	741,716	1,066 (0.14)	
Age group, y			<0.01
<18	84,025	67 (0.08)	
18–44	625,865	916 (0.15)	
45–59	375,826	697 (0.19)	
60–74	248,057	387 (0.16)	
≥75	89,325	95 (0.11)	
Socioeconomic status			<0.01
Low	228,613	106 (0.05)	
Middle	706,198	976 (0.14)	
High	488,287	106 (0.05)	
Heart disease			0.03
No	1,348,587	2,026 (0.15)	
Yes	74,511	136 (0.18)	
Diabetes			0.47
No	1,299,843	1,982 (0.15)	
Yes	123,255	177 (0.14)	
Hypertension			0.22
No	1,149,913	1,770 (0.15)	
Yes	273,185	392 (0.14)	
Chronic kidney disease			0.01
No	1,355,164	2,083 (0.15)	
Yes	67,934	79 (0.12)	
Immunosuppressive disorder			0.76
No	1,406,560	2,139 (0.15)	
Yes	16,538	23 (0.14)	

*By χ^2/Fisher exact test statistic.
